# Human and environmental impacts of nanoparticles: a scoping review of the current literature

**DOI:** 10.1186/s12889-023-15958-4

**Published:** 2023-06-03

**Authors:** Elizabeth Adjoa Kumah, Raoul Djou Fopa, Saeed Harati, Paul Boadu, Fatemeh Vida Zohoori, Tannaz Pak

**Affiliations:** 1 Depeartment of International Public Health, Liverpool School of Tropical Medicine, Liverpool, UK; 2 School of Computing, Engineering & Digital Technologies, Teesside University, Middlesbrough, TS1 3BX UK; 3 Department of Health Services Research and Policy, London School of Hygiene and Tropical Medicine, London, UK; 4 School of Health and Life Sciences, Teesside University, Middlesbrough, UK

**Keywords:** Biomarkers, Cytotoxicity; Environmental health, Genotoxicity, Human health, Impact, Nanoparticles, Scoping review, Toxicity

## Abstract

Use of nanoparticles have established benefits in a wide range of applications, however, the effects of exposure to nanoparticles on health and the environmental risks associated with the production and use of nanoparticles are less well-established. The present study addresses this gap in knowledge by examining, through a scoping review of the current literature, the effects of nanoparticles on human health and the environment. We searched relevant databases including Medline, Web of Science, ScienceDirect, Scopus, CINAHL, Embase, and SAGE journals, as well as Google, Google Scholar, and grey literature from June 2021 to July 2021. After removing duplicate articles, the title and abstracts of 1495 articles were first screened followed by the full-texts of 249 studies, and this resulted in the inclusion of 117 studies in the presented review.

In this contribution we conclude that while nanoparticles offer distinct benefits in a range of applications, they pose significant threats to humans and the environment. Using several biological models and biomarkers, the included studies revealed the toxic effects of nanoparticles (mainly zinc oxide, silicon dioxide, titanium dioxide, silver, and carbon nanotubes) to include cell death, production of oxidative stress, DNA damage, apoptosis, and induction of inflammatory responses. Most of the included studies (65.81%) investigated inorganic-based nanoparticles. In terms of biomarkers, most studies (76.9%) used immortalised cell lines, whiles 18.8% used primary cells as the biomarker for assessing human health effect of nanoparticles. Biomarkers that were used for assessing environmental impact of nanoparticles included soil samples and soybean seeds, zebrafish larvae, fish, and Daphnia magna neonates.

From the studies included in this work the United States recorded the highest number of publications (*n* = 30, 25.64%), followed by China, India, and Saudi Arabia recording the same number of publications (*n *= 8 each), with 95.75% of the studies published from the year 2009. The majority of the included studies (93.16%) assessed impact of nanoparticles on human health, and 95.7% used experimental study design. This shows a clear gap exists in examining the impact of nanoparticles on the environment.

**Supplementary Information:**

The online version contains supplementary material available at 10.1186/s12889-023-15958-4.

## Highlights

• While nanoparticles are beneficial in a range of applications, they pose significant threats to humans and the environment.

• Immortalised cell lines are mostly used as biomarkers to assess human health effect of nanoparticles.

• Biomarkers such as soil samples and zebrafish larvae are used to investigate the environmental effect of nanoparticles.

• This work has revealed the toxic effects of nanoparticles to include production of oxidative stress, DNA damage, apoptosis, cell death, and induction of inflammatory responses.

**Supplementary Information:**

The online version contains supplementary material available at 10.1186/s12889-023-15958-4.

## Introduction

### Importance and meaning

Nanoparticles are small particles ranging from 1 to 100 nm (nm) in size [1]. They are used in a wide range of applications and can be grouped into four types: 1) inorganic-based nanoparticles, 2) carbon-based nanoparticles, 3) organic/polymer nanoparticles, and 4) composite-based nanoparticles [2]. Inorganic-based nanoparticles are made up of different metal and metal oxides. Examples of metal-based inorganic nanoparticles include aluminium, silver, gold, zinc, lead, iron, cadmium, and copper, whereas examples of metal oxide-based inorganic nanoparticles include aluminium oxide, copper oxide, iron oxide, silica, zinc oxide, titanium oxide, and magnesium aluminium oxide. Carbon-based nanoparticles include fullerene, graphene, multi- and single-walled carbon nanotubes, carbon black, and carbon fibres. Organic-based nanoparticles are derived from organic materials without carbon, for example, liposome, dendrimers, cyclodextrin, and micelle, whereas composite nanoparticles are made from combinations of metal oxide-based, metal-based, organic-based, and/or carbon-based nanoparticles.

In recent years, nanoparticles have gained increasing attention due to their use in consumer products, medicine, soil, and aquatic environments. For example, nanoparticles have been used for textiles [3], water treatment [4], environmental remediation [5–7], cancer therapy [8], radiology [9], and cosmetics [10]. This growing attention and extensive usage of nanoparticles is due to specific novel characteristics exhibited by such particles, which results from their small size and large surface area [11]. These unique qualities, while advantageous, pose certain risks to living organisms.

### The harmful effects of nanoparticles

The small sizes of nanoparticles give them the ability to permeate physiological barriers of living organisms, causing harmful biological reactions. Nanoparticles are known to enter the human body through the lung, intestinal tract, or skin, and can be toxic to the brain, cause lung inflammation and cardiac problems [12]. In fact, certain nanoparticles have been found to cause permanent cell damage through organ injury and oxidative stress, due to their size and composition. In a study by Magrez et al. [13] to assess the toxic effect of carbon-based nanoparticles on lung cancer cells, the authors reported findings suggesting that carbon-based nanoparticles cause size-dependent cytotoxicity. The level of toxicity of nanoparticles is suggested to be dependent on factors such as composition of the nanoparticle, size, surface functionality, crystallinity, and aggregation [14]. Moreover, the toxicity of a nanoparticle in an individual is dependent on the genetic make-up of that individual, which is determined by the individual's ability to adapt and respond to toxic substances.

### The gaps of previous studies

There are growing concerns regarding the toxicologic effects of nanoparticles, and frequent exposure to nanoparticles is regarded as a public health threat [15]. While there is extensive evidence about the benefits of nanoparticles, as well as the potential health and environmental risks associated with its production and use, current understanding of the impact of nanoparticles exposure to human health and the environment is limited. The current review seeks to explore, through a scoping review of the current literature, the effects of nanoparticles on human health and the environment. This review is unique as it adopts a systematic scoping approach to explore the current literature on the health risks posed by the manufacture, distribution, and use of nanoparticles. Published studies in this area have mainly used a narrative literature review approach [2, 16, 17].

### Objective and research questions

The objective of this review is to map the distribution of the current literature on the human and environmental impacts of nanotoxicity. Specifically, this scoping review will be guided by the following research questions:

1. What is the relative distribution of the current literature on the human and environmental impact of nanotoxicity?
2. Which exposure pathways and nanoparticles have been researched and which have not?
3. What biomarkers have been used in assessing the human and environmental impact of exposure to nanoparticles?

## Methods

This scoping review was conducted and reported in accordance with the Joanna Briggs Institute Reviewers Manual [18]. The following steps were followed:

1. Defining and aligning the objectives and research questions
2. Developing and aligning the inclusion criteria with the objectives and research questions
3. Describing the planned approach to evidence searching and selection
4. Searching for the evidence
5. Extracting the evidence
6. Charting the evidence
7. Summarising the evidence in relation to the objectives and research questions

The Preferred Reporting of Items for Systematic Reviews and Meta-Analysis (PRISMA) statement was used to summarise the screening process. The protocol of this review has been registered with the Open Science Framework [19].

### Search strategy

The aim of the search strategy was to find both published and unpublished studies that have examined the effect of nanotoxicity on human health and the environment. Search terms consisted of a combination of key terms and concepts in the objective and research questions, using the Boolean operators, 'AND', and 'OR' as follows:

(nanomaterials OR nanoparticles OR nanostructures) AND (toxicity OR health) AND (“biomarker* of exposure” OR biomarker OR exposure) AND (human OR environment).

The search was limited to peer-reviewed articles published from the year 2000. This was to enable us to study the current literature (research conducted over the last 2 decades). The search was limited to primary studies published in the English language due to difficulties with language translation.

Table 1 below presents a list of the databases, grey literature, and search engines that were searched for eligible papers. The reference list of all included papers was also searched for additional papers on the subject matter.

**Table 1:** Sources of literature

Database sources	Grey Literature	Search Engines
ScienceDirect Sage Journals Online Campbell Collaboration Cochrane collaboration Embase Medline CINAHL Web of Science Scopus	OpenGrey NICE Evidence Search The Grey Literature Report Bielefeld Academic Search Engine (BASE) Australian Bureau of Statistics (ABS)	Google Google Scholar

For the database searches, a master search strategy was first developed using the Medline database, this was then modified for the other databases. The supplementary material file presents the Medline search history. The literature search was conducted between 1^st^ June 2021 and 31^st^ July 2021.

### Reference management

All search results were imported into an Endnote library to help manage references and to remove duplicate articles. Once duplicates were removed, the search results were exported from Endnote into Covidence (a web-based software platform that streamlines the production of scoping/systematic reviews) for screening. The Covidence software was also useful in identifying and deduplicating articles that could not be identified by Endnote.

### Selection criteria

The following criteria were used to identify eligible articles for inclusion in the review.

#### Inclusion criteria

##### Types of participants

Studies that have assessed the human and environmental impacts of nanotoxicity were considered for inclusion in this review. Human participants included children and/or adults of any age, gender, or ethnicity. Studies involving the use of animals as biomarkers for assessing the environmental impact of nanotoxicity were also considered for inclusion.

##### Concept

Studies that have examined the impacts of nanotoxicity as well as the biomarkers for assessing exposure to nanoparticles were eligible for inclusion in this review. While all types of nanoparticles were considered for inclusion, attention was given to studies involving metallic (oxides, pure metal) and carbonaceous (fullerenes, carbon nanotubes, and graphene) nanoparticles. This is mainly due to these particles being widely produced and used [20], therefore, they are considered the most relevant for public health.

##### Context

Studies from any geographical location aimed at assessing the human and/or environmental impact of nanotoxicity were considered eligible for inclusion. Studies whose full texts were in a language other than English were excluded because there were no available translators.

##### Study types

We included all original primary research (both quantitative and qualitative), including, but not limited to randomised controlled studies, quasi-experimental studies, surveys, retrospective and prospective cohort studies, case studies, and phenomenological studies.

#### Exclusion criteria

The following exclusion criteria were applied to the title and abstract, as well as the full-text review stage:

- Irrelevant problem/focus: studies that have not examined the human and/or environmental impact of nanotoxicity, or the biomarkers for assessing exposure to nanoparticles
- Irrelevant type of study: review reports or studies that did not contain any original research

### Selection of studies

We employed a two-step screening process to assess search results for eligible studies. The first level involved screening of the titles and abstracts and was done independently by two reviewers (EK and RF). The next step was carried out independently by three reviewers (EK, RF, and SH) and involved screening of the full-texts of potentially eligible papers. Disagreements between reviewers were resolved through discussions and consensus. Where disagreements persisted, a third reviewer (TP or FVZ) was consulted.

### Data charting

We developed a standardised data extraction form in the Covidence software for data extraction. The form was designed to collect the following information from included studies: year of publication, aim/objective of study, study design, country, type of nanoparticle, application of the nanoparticle, major exposure route(s), biomarker/model used, how biomarker was obtained, and study outcome(s).

The developed data extraction form was pilot-tested using 10% of the included articles before beginning the actual data extraction. Data extraction was done by one reviewer (PB, RF, or SH) and verified by another (EK, TP, or FVZ), using the Covidence software.

### Data synthesis

The extracted data was first exported into Excel for editing and to check for accuracy. The edited data was then exported from Excel into SPSS (version 26) to aid with data synthesis. Descriptive statistics was used to report included studies by their characteristics and outcome measures, described below.

#### Characteristics of included studies

- *Year of publication:* studies were grouped based on their year of publication*.* As stated earlier, this included studies published from the year 2000 to July 2021 (the date of completion of literature searches).
- *Country in which study was conducted*: to assess the distribution of the current literature on human and environmental impact of nanotoxicity, the countries in which eligible studies were conducted were classified into six regions based on the World Bank’s classification of countries. This included: East Asia and Pacific, Europe and Central Asia, Latin America and Caribbean, Middle East and North Africa, North America, South Asia, and Sub-Saharan Africa (World Bank Group, 2018).
- *Study design*: randomised controlled trial, non-randomised controlled trial, cohort study, experimental study, case control study, longitudinal study, uncontrolled before and after studies.
- *Impact/effect assessed*: human health and/or environment

#### Outcome measures

- *Type of nanoparticle*: This was divided into four groups: 1) inorganic-based nanoparticles, 2) carbon-based nanoparticles, 3) organic nanoparticles, and 4) composite-based nanoparticles two groups, metallic (oxides, pure metal) and carbonaceous (fullerenes and carbon nanotubes) particles.
- *Biomarker or model used in assessing human and/or environmental exposure*: primary cell or immortalised cell line
- *Effect/impact on human health and/or the environment*

A narrative synthesis was then used to further explore findings.

## Results

### Search results

**Fig. 1:**
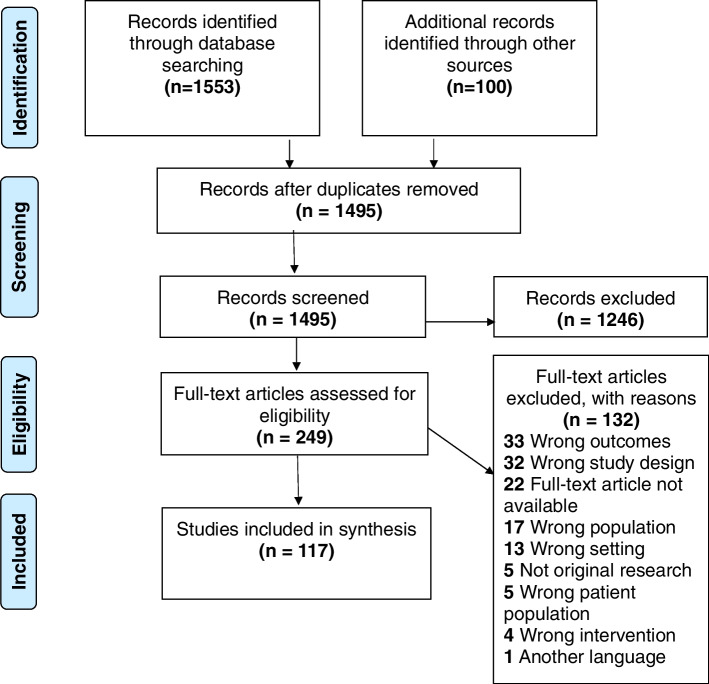
Study flow diagram (adapted from Moher et al., 2009)

The database searches resulted in 1553 papers (presented in Fig. 1): Medline (*n* = 1,381); ScienceDirect (*n* = 0); Sage Journals Online (*n* = 50); Campbell Collaboration (*n* = 0); Cochrane Collaboration (*n* = 0); Embase (*n* = 5); Scopus (*n* = 6); Web of Science (*n* = 50); CINAHL (*n* = 61). Google and Google Scholar searches yielded 100 results, and no article was obtained from grey literature searches. Following removal of duplicate articles, the titles and abstracts of 1495 articles were screened to assess their eligibility for inclusion, which resulted in the exclusion of a total of 1246 articles as they did not meet the inclusion criteria. As such, the full texts of 249 articles were assessed for eligibility. Following this stage, a total of 132 articles were excluded for several reasons (see Fig. 1), whereas 117 studies qualified for inclusion in the review.

### Characteristics of included studies

**Fig. 2:**
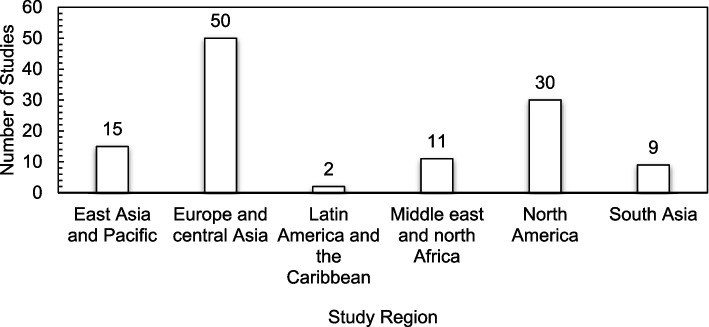
Classification of Studies by Region

The studies included in this review originated from 23 countries across several continents, with the majority of the studies originating from Europe and Central Asia (*n* = 50). Nevertheless, the United Sates recorded the highest number of publications (*n* = 30), followed by China, India, and Saudi Arabia recording the same number of publications (*n* = 8). The lowest number of studies (*n* = 1 each) originated from Argentina, Czech Republic, Egypt, Mexico, Pakistan, Poland, and Russia. There were no studies recorded from Sub-Saharan Africa. Figure 2 presents a classification of the included studies by region.

Included studies were published between the year 2006 and 2021, with a high proportion of the articles (95.75%) published from the year 2009. However, the year 2020 recorded the highest number of publications (*n* = 15; 12.82%), followed by 2016 (*n* = 14; 11.97%). Table 2 below presents the number of publications per year.

**Table 2:** Number of publications per year (*n* = 117)

Year	Number	Percent
2006	1	0.85
2007	1	0.85
2008	3	2.56
2009	10	8.55
2010	5	4.27
2011	6	5.13
2012	11	9.40
2013	6	5.13
2014	10	8.55
2015	5	4.27
2016	14	11.97
2017	6	5.13
2018	9	7.69
2019	8	6.84
2020	15	12.82
2021	7	5.98
**Total**	**117**	**100**

**Fig. 3:**
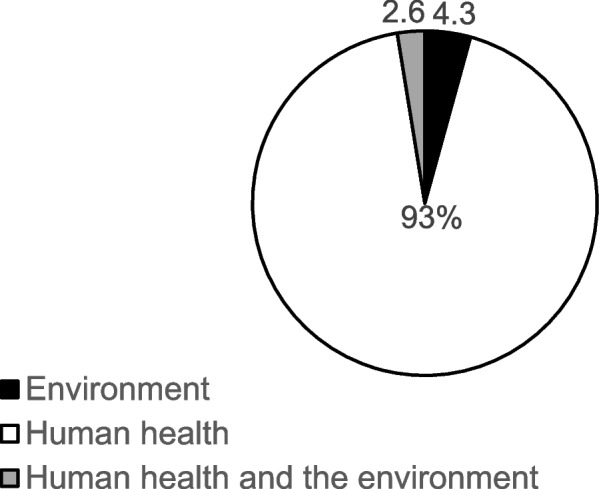
Effect/impact of nanoparticles on human/environmental health

The majority of the studies used an experimental study design (*n* = 112, 95.7%), with only 5 (4.3%) studies employing a cross-sectional design. Regarding the type of impact/effect of nanoparticle assessed, a vast majority of the studies assessed impact on human health (*n* = 109), 5 of the studies assessed effects on the environment, with only 3 studies assessing both human and environmental health impact (Fig. 3).

### Outcome measures

Just over 65% (*n* = 77) of the included studies investigated the human and/or environmental effect of inorganic-based nanoparticles. The inorganic-based nanoparticles that were investigated include, but not limited to, bismuth oxide (Bi_2_O_3_), silicon dioxide (SiO_2_), copper oxide (CuO), zinc oxide (ZnO), titanium dioxide (TiO_2_), silver (Ag), gold (Au), platinum (Pt), iron oxide (Fe_2_O_3_), cerium oxide (CeO_2_), cobalt oxide (Co_3_O_4_), aluminium oxide (Al_2_O_3_), molybdenum trioxide (MoO3), magnesium oxide (MgO), nickel oxide (NiO), chromium oxide (Cr_2_O_3_), tungsten oxide (WO_3_), yttrium oxide (Y_2_O_3_), and manganese oxide (Mn_2_O_3_).

Thirty-five (29.9%) studies reported on carbon-based nanoparticles (including single and multi-walled carbon nanotubes (SWCNTs/MWCNTs), graphene oxide (GO), and graphene nanoplatelets, GNP). Three studies [21–23] reported on both inorganic- and carbon-based nanoparticles; one study [24] reported on both inorganic-based and polymer nanoparticles (i.e., Titanium dioxide, terbium-doped gadolinium, and polylactic-co-glycolic acid, PLGA), whereas another study [25] investigated the effect of Poly lactic-co-glycolic acid (a polymer) nanoparticle on the environment.

The most investigated nanoparticles were ZnO (*n* = 25), followed by MWCNTs (*n* = 20), TiO_2_ (*n* = 16), CeO (*n* = 15), SWCNTs and Fe_2_O_3_ (*n* = 14), and SiO_2_ (*n* = 12). The least studied nanoparticles include Pt, Au, MgO, MoO_3_, WO_3_, Carbon Black (CB), and GNP with only one report available.

A significant number (*n* = 90, 76.9%) of the included studies used immortalised cell lines as the biomarker for assessing the human health effect of nanoparticles. Examples of the immortalised cell lines that were used include the human hepatocarcinoma cell line (HepG2), human (alveolar) epithelial A549 cell line with human monocyte-derived dendritic cells (MDDCs) and macrophages (MDMs), Melanoma cells and human foreskin fibroblasts, human airway epithelial (BEAS-2B) cells, human bronchial epithelium (BEAS-2B) cells, human neuroblastoma SHSY5Y cell line, human keratinocyte (HaCaT) cell line, and MCF-7 cell line, which is a human breast cancer cell line with oestrogen, progesterone and glucocorticoid receptors. Immortalised cell lines were mostly purchased/obtained from organisations such as the American Type Culture Collection (ATCC, Manassas, VA, USA).

Twenty-two studies used primary cells obtained from study participants/volunteers. Examples of the primary cells that were used as biomarkers by included studies are human bone marrow mesenchymal stem cells (hBMMSCs) taken from the iliac crest of human donors, human lymphocytes (blood), and human dermal fibroblasts which were isolated by the outgrowth method using infant foreskins obtained after circumcision. Workplace air samples have also been used to investigate workplace exposures to graphene nanoplatelets [26]. Five studies [25, 27–30] that reported on the environmental effect of nanoparticles used a variety of biomarkers, including soil samples and soybean seeds, Allium cepa bulbs, zebrafish larvae, seedlings of buckwheat, Nitrosomonas europaea KCTC 12270 bacterium (an ammonia-oxidizing bacterium) and Nitrospira moscoviensis (a nitrite-oxidizing bacterium), as well as aquatic species including Daphnia magna neonates, fish, and Carp (Cyprius carpio). The studies included in this review reported several toxicities associated with the production and application of nanoparticles. The most reported health impact of nanoparticles was found to be decreased cell viability and/or cell death (observed by twenty-nine studies). Twenty-eight studies also noted reactive oxygen species generation as a result of exposure to nanoparticles, especially to CNT (*n* = 7), ZnO (*n* = 7), SiO_2_ (*n* = 5), and TiO_2_ (*n* = 4). The third commonly observed health impact was dose-dependent oxidative stress in the biomarkers (*n* = 25), particularly, in cases of exposure to SiO_2_ (*n* = 5), ZnO (*n* = 5), Fe_3_O_4_ (*n* = 4), CeO_2_ (*n* = 3), and CuO (*n* = 3). In addition, there were sixteen reports regarding DNA damage after exposure to nanoparticles, mainly for ZnO (*n* = 4) and MWCNTs (*n* = 3). Table 3 presents a comprehensive outline of the effects (human health and the environment) reported by each of the included studies. These are further explored in the ensuing section.

**Table 3:** Studies on effect (human health and environment) of various types of nanoparticles

Reference	Type of nanoparticle	Biomarker/model used	Toxicity/harmful impacts of nanoparticle
**Inorganic-based Nanoparticles**			
Ahamed et al. [31]	Bismuth oxide (Bi_2_O_3_)	MCF-7 cell line (a human breast cancer cell line with estrogen, progesterone, and glucocorticoid receptors)	• Reduces cell viability • Induces membrane damage Dose-dependently • Oxidative stress • Reactive Oxygen Species generation
Eom & Choi [32]	Fumed and porous Silicon dioxide (SiO_2_)	Human bronchial epithelial cell (Beas-2)	• Oxidative stress • Induction of heme oxygenase-1 (HO-1)
Bengalli et al. [33]	Copper (CuO) and Zinc oxide (ZnO)	Reconstructed human epidermis model and fibroblast monolayer	• Deeply affect the epidermal tissue and the underlying dermal cells upon trans-epidermal permeation
Ferraro et al. [34]	Titanium dioxide (TiO_2_)	Human neuroblastoma (SH-SY5Y) cell line	• Reactive Oxygen Species generation • Apoptosis • Induction of endoplasmic (ER) stress • Neurotoxicity
Gambardella et al. [35]	Silicon dioxide (SiO_2_)	Sea urchin Paracentrotus lividus sperms	• Reduced toxicity • Neurotoxic damage • Decrease of acetylcholinesterase (AChE) expression • No effect on fertilization capability • Induced of toxic effect on the offspring
Yusefi-Tanha et al. [30]	Zinc oxide (ZnO)	Soil samples and Soybean seeds	• Oxidative stress • Significant particle size-, morphology-, and concentration-dependent influence on seed yield and lipid peroxidation
Oh et al. [36]	Citrate-coated silver (Ag)	Human embryonic stem cell (hESC)	• Oxidative stress • Dysfunctional neurogenesis
Zhao et al. [37]	Silicon dioxide (SiO_2_)	Lung bronchial epithelial cells (BEAS-2B)	• Disturbs global metabolism • Oxidative stress • Generation of reactive oxygen species • Significantly perturbs mitochondrial dysfunction-related GSH metabolism and pantothenate and coenzyme A (CoA) biosynthesis • Causes abnormality in mitochondrial structure and mitochondrial dysfunction
Ying et al. [38]	Superparamagnetic Iron oxide (SPIO)	A3 human T lymphocytes	• Causes concentration-dependent nanotoxicity
Jing et al. [39]	Copper oxide (CuO)	Lung adenocarcinoma cells (A549 cells) and human bronchial epithelial cells (HBEC)	• Significantly reduced cell viability • Increased lactate dehydrogenase (LDH) release • Reactive Oxygen species and IL-8 generation
Lojk et al. [40]	Silicon dioxide (SiO_2_), Titanium dioxide (TiO_2_), Silver (Ag), Polyacrylic acid (PAA) coated cobalt ferrite (CoFe_2_O_4_)	Human neuroblastoma (SH-SY5Y) cell	• Neurotoxicity • Reactive Oxygen species formation • Membrane damage • Autophagy dysfunction for TiO2 P25 NPs • Decrease of cell viability for TiO2 FG NPs
Tsai et al. [41]	Titanium dioxide (TiO_2_) and Cerium dioxide (CeO_2_)	BEAS-2B epithelial cell	• TiO_2_ induces apoptosis and hypersecretion of mucus • CeO_2_ NPs reduce cytosolic Ca^2+^ changes and mitochondrial damage caused by TiO_2_ NPs
Sramkova et al. [42]	Titanium dioxide (TiO_2_), Silicon dioxide (SiO_2_), magnetite (Fe_3_O_4_) and gold (Au)	Human renal proximal tubule epithelial TH1 cell line	• None of the NPs induced any DNA strand breaks and oxidative DNA lesions regardless of the exposure (static and dynamic conditions) • No cytotoxicity was observed, except for Fe_3_O_4_NPs
Bell et al. [43]	Silicon dioxide (SiO_2_)	SH-SY5Y human neuroblastoma (ATCC CRL-2266) Human epithelial type-2 (HEp-2) cells (ATCC CCL-23)	• Destabilizes mitochondrial membrane potential • stimulates reactive oxygen species production • Promotes cytotoxicity
Akhtar et al. [44]	Silicon dioxide (SiO_2_)	Human lung epithelial cells (A549 cells)	• Lower concentration: o Induction of reactive oxygen species o Membrane damage • Higher concentration: o Reactive oxygen species generation o GSH depletion
Gaiser et al. [27]	Silver (Ag) and cerium oxide (CeO_2_)	Aquatic species: Daphnia magna neonates, fish, Carp (Cyprius carpio) Human model: C3A human hepatocyte cell line and caco-2 human intestinal epithelial cells	• Ag is more cytotoxic than CeO_2_ • Both particles when in diet have the potential to enter the body following ingestion
Dankers et al. [45]	CeO_2_, Mn_2_O_3_, CuO, ZnO, Co_3_O_4_, and WO_3_	Lung epithelium and dendritic cells	• Metal oxide NPs elicit minimal proinflammatory effects
Chen et al. [46]	Zinc oxide (ZnO)	Human umbilical vein endothelial cells (HUVECs)	• ZnO induces significant cellular ER stress • Higher doses of ZnO induces apoptosis
Patil et al. [47]	Titanium dioxide (TiO_2_) and zinc oxide (ZnO)	Lung fibroblast (MRC5)	• Impedes genomic DNA hypomethylation
Mohamed et al. [48]	Silicon dioxide (SiO_2_)	Human monocytic leukemia cell line THP-1 and human alveolar epithelial (A549) cell line	• Low degree of cytotoxicity at all concentrations • Stress-related cellular response at high concentrations
Mancuso & Cao [14]	Copper oxide (CuO)	Human bone marrow mesenchymal stem cells (hBMMSCs)	• Bigger sizes exhibit significant cytotoxicity at all concentrations • Micro-sized particles exhibit very low cytotoxicity at the same concentration
Alshatwi et al. [49]	Aluminium oxide (Al_2_O_3_)	Human mesenchymal stem cells (hMSCs)	• Dose-dependent decreased cell viability • Decreased mitochondrial membrane potential with increasing concentrations after 24 exposures • Down-regulation in the expression of the antioxidant enzyme SOD • Did not induce apoptosis • Dose-dependent oxidative stress
Baber et al. [50]	Two amorphous silica coated (MagSilica 85, MagSilica 50) and uncoated iron oxide NPs (Fe_3_O_4_)	BEAS-2B (immortalized normal human bronchial epithelium)	• Little to no indications of cytotoxicity • No induction of inflammatory response/oxidative stress
Corbalan et al. [51]	Amorphous Silicon dioxide (SiO_2_)	Blood	• Induced an upregulation of selectin P expression and glycoprotein IIb/IIIa activation on the platelet surface membrane • Platelet aggregation
Branica et al. [52]	Zinc oxide (ZnO)	Blood (Human lymphocyte)	• Higher concentrations increase cytogenetic damage and intracellular Zn^2+^ levels in lymphocytes
Gurunathan et al. [53]	Platinum (Pt)	Human acute monocytic leukemia (THP-1) macrophages	• Decreased cell viability and proliferation • Induces cell death • Oxidative stress • Mitochondrial dysfunction • Endoplasmic reticulum stress (ERS) • Proinflammatory responses
Hussain et al. [54]	Cerium dioxide (CeO_2_)	Human peripheral blood monocytes	• CeO_2_ NPs at non-cytotoxic concentrations neither modulate pre-existing inflammation nor prime for subsequent exposure to lipopolysaccharides in human monocytes from healthy subjects
Zerboni et al. [55]	Zinc oxide (ZnO) and Cupper oxide (CuO)	Human alveolar epithelial cells, A549	• The presence of diesel exhaust particles (DEP) introduces new physicochemical interactions able to increase the cytotoxicity of ZnO and to reduce that of CuO NPs
Zielinska et al. [56]	Silver (Ag)	Human fetal osteoblast cells (hFOB 1.19)	• Cell death • Reactive oxygen species production
Rajiv et al. [57]	Cobalt (II, III) oxide (Co_3_O_4_); Iron (III) oxide (Fe_2_O_3_), Silicon dioxide (SiO_2_), and Aluminium oxide (Al_2_O_3_)	Human lymphocytes	• Co_3_O_4_ NPs showed a decrease in cellular viability and an increase in cell membrane damage followed by Fe_2_O_3_, SiO_2_, and Al_2_O_3_ NPs in a dose-dependent manner • Oxidative stress • Lipid peroxidation • Depletion of catalase • Reduced glutathione • Superoxide dismutase
Alarifi et al. [58]	Copper oxide (CuO)	Human skin epidermal cell line (HaCaT; passage no. 20)	• Decrease in cell viability • Reduction in glutathione and induction in lipid peroxidation, catalase, and superoxide dismutase • Apoptosis • Necrosis • Induces DNA damage mediated by oxidative stress
Sun et al. [59]	Zinc oxide (ZnO), Fe_2_O_3_, Iron (II, III) oxide (Fe_3_O_4_), Magnesium oxide (MgO), Aluminium oxide (Al_2_O_3_), Copper (II) oxide (CuO)	Human cardiac microvascular endothelial cells (HCMECs)	• Fe_2_O_3_, Fe_3_O_4_, and Al_2_O_3_ NPs did not have significant effects on cytotoxicity, permeability, and inflammation response • ZnO, CuO, and MgO NPs produced cytotoxicity at a concentration-dependent and time-dependent manner and elicited permeability and inflammation response in HCMECs
Tolliver et al. [60]	Titanium dioxide – (TiO_2_), Zinc oxide – (ZnO), Copper oxide – (CuO), Manganese oxide (Mn_2_O_3_), Iron oxide – (Fe_2_O_3_), Nickel oxide – (NiO), Chromium oxide – (Cr_2_O_3_)	Human lung cancer cell model (A549)	• All NPs aside from Cr_2_O_3_ and Fe_2_O_3_ showed a time- and dose-dependent decrease in viability • All NPs significantly inhibited cellular proliferation • Apoptosis • Cell cycle alteration in the most toxic NPs
Rothen-Rutishauser et al. [61]	Cerium oxide (CeO_2_)	Adenocarcinomic human alveolar basal epithelial (A549) cell line	• Generation of oxidative DNA damage • Causes tightness of the lung cell monolayer • Dose-dependent cellular response
Benameur et al. [62]	Cerium Oxide (CeO_2_)	Human dermal fibroblasts	• Genotoxicity • Reactive oxygen species production • Lower doses of CeO_2_ did not induce significant cytotoxicity • Induces lipid peroxidation and decline of cellular glutathione level at concentrations above 0.00006 M
Gojova et al. [63]	Cerium oxide (CeO_2_)	Human aortic endothelial cells (HAECs)	• Causes very little inflammatory response even at higher doses
Lee et al. [29]	Zinc oxide (ZnO)	Natural soil and seedlings of buckwheat	• The effect of ZnO NPs on soil bacterial depends on the presence of plants • The soil–plant interactive system helps to decrease the toxic effects of ZnO nanoparticles on the rhizobacteria population relative to soil systems not containing plants
Hildebrand et al. [64]	Magnetite (Fe_3_O_4_) and palladium magnetite (Pd/Fe_3_O_4_)	1) Human cell lines: Colon adenocarcinoma cells, CaCo-2 (HTB-37), Human keratinocyte cells, (HaCaT) 2) Fish cell line: Rainbow trout gills (RTgill-W1) cell line	• No initiation of reactive oxygen species production • Little impact on the viability of colon adenocarcinoma cells, human keratinocyte cells, and the rainbow trout gills cell line • No toxic effect was found
Lai et al. [65]	Titanium dioxide (TiO_2_)	Human astrocytoma and human fibroblasts	• Induces cell death • Apoptosis • Necrosis
Ahamed et al. [66]	Copper oxide (CuO)	Human lung epithelial cells (A 459)	• Dose-dependent reduction in cell viability • Induces oxidative stress • Depletion of glutathione • Induction of lipid peroxidation, Catalase and superoxide dismutase • Induces cellular damage (indicated by the expression of Hsp70, the first tier biomarker of cellular damage)
Vergaro et al. [67]	Titanium dioxide (TiO_2_)	Human bronchial epithelial cells (BEAS-2B)	• Induces a low photo reactivity and a toxic effect lower than Aeroxide P25 of the nano-TiO_2_ powders
Dávila-Grana et al. [68]	Zinc oxide (ZnO), Titanium dioxide (TiO_2_), Cerium dioxide (CeO_2_), Aluminium oxide (Al_2_O_3_), Yttrium (III) oxide (Y_2_O_3_)	Jurkat cell line	• The combination of nanoparticles induces changes in cell signalling mediated by the MAPKs and nuclear factor-κB (NF-κB) • Al_2_O_3_ NPs had a protective effect when combined with the ZnO NPs • CeO_2_ and Y_2_O_3_ Nps induced a synergistic effect on the toxicity and p38 activation • TiO_2_ nanoparticles increase the toxicity induced by ZnO nanoparticles but reduced the phosphorylation of the signalling proteins
Pierscionek et al. [69]	Cerium oxide (CeO_2_)	Human lens epithelial cells	• Epithelial cells can sustain normal growth when exposed to lower concentrations of nanoceria • Induces genotoxicity when exposed for longer periods
Rafieepour et al. [70]	Magnetite iron oxide (Fe_3_O_4_), polymorphous silicon dioxide (P-SiO_2_)	Adenocarcinomic human alveolar basal epithelial (A549) cell line	• Reduces cell viability • Reduces cellular glutathione content and mitochondrial membrane potential • Increases reactive oxygen species generation in both single and combined exposures of Fe_3_O_4_ and P-SiO_2_ • The toxic effects of combined exposure to these NPs were less than the single exposures
Ickrath et al. [71]	Zinc oxide (ZnO)	Human mesenchymal stem cells (hMSC)	• Induces cytotoxic effect at higher concentrations of 50mcg/mL • Induces genotoxic effects in hMSC exposed to between 1 and 10mcg/mL ZnO-NP
Radeloff et al. [72]	Iron oxide (Fe_3_O_4_)	Human adipose tissue derived stromal cells (hASCs)	• No effect on the physiological functions of human adipose tissue derived stromal cell (hASCs)
Jin et al. [73]	Zinc oxide (ZnO)	Zebrafish larvae and human neuroblastoma cells SH-SY5Y	• Smaller sizes of ZnO showed slightly higher toxicity than the larger sizes • Long ZnO NRs (l-ZnO NRs) harbours a remarkably potential risk for the onset and development of Parkinson’s disease at relatively high doses
Kumari et al. [74]	Cerium oxide (CeO_2_)	Human neuroblastoma cell line (IMR32)	• Induces size- and dose-dependent toxicity (oxidative stress and genotoxicity) • CeO_2_ did not induce toxicity below 100 mg/mL concentration • IMR32 cells are less sensitive to CeO_2_ NPs
Fernández-Bertólez et al. [75]	Silica-coated iron oxide nanoparticles (SiO_2_)	Human glioblastoma A172 cells	• Cytotoxicity (cell cycle disruption and cell death induction) • Rarely induces genotoxic effects • No alteration in the DNA repair process
Gliga et al. [76]	Nickel (Ni), nickel oxide (NiO)	Human bronchial epithelial cell line (BEAS-2B)	• Long-term exposures (six weeks) changes gene expressions • Induces DNA strand breaks and alter cell cycle after six weeks of repeated exposure • Nickel causes no effect on cell transformation (ability to form colonies in soft agar) or cell motility
Kennedy et al. [77]	Iron oxide (Fe_3_O_4_), zinc oxide (ZnO), Yttrium oxide (Y_2_O_3_), and cerium oxide (CeO_2_)	Human aortic endothelial cells (HAECs)	• Induces oxidative stress • Zinc oxide more toxic than yttrium oxide • No effect on HAECs when exposed to Iron oxide and cerium oxide
Schanen et al. [78]	Titanium dioxide and cerium dioxide (TiO_2_ and CeO_2_)	PBMC blood product	• Low dose exposures modulate human innate and adaptive immunity (i.e., dendritic cells activation and primary CD4 T helper cell differentiation state)
Seker et al. [79]	Zinc oxide (ZnO)	Human periodontal ligament fibroblast cells (hPDLFs)	• Causes cell index decrease at concentrations of 50 to 100lg/mL • Induces changes in cell morphology • Induces harmful effects on cell viability and membrane integrity • Necrosis • Cell death (in terms of morphological change or cellular shrinkage) at doses higher than 50lg/mL • Cytotoxicity depends on duration of exposure and concentration
Könen-Adıgüzel & Ergenel [80]	Cerium dioxide (CeO_2_)	Human blood lymphocytes	• Induces genotoxicity even at 3–24 h exposure under in vitro conditions
Henson et al. [81]	Cupric (II) oxide (CuO), Polyvinylpyrrolidone (PVP) coated NPs	A three-dimensional model of the human small intestine, EpiIntestinalTM(SMI-100)	• Induces dose- and time-dependent viability of human cells
Gojova et al. [82]	Iron oxide (Fe_2_O_3_), Yttrium oxide (Y_2_O_3_), and Zinc oxide (ZnO)	Human aortic endothelial cells (HAECs)	• All three types of nanoparticles are internalized into HAECs and are often found within intracellular vesicles • No inflammatory response after exposure to Fe_2_O_3_ • Y_2_O_3_ and ZnO nanoparticles elicit pronounced inflammatory response
Alarifi et al. [83]	Zinc Oxide (ZnO)	Human skin melanoma (A375) cells	• Decrease in cell viability • Causes morphological changes • Induces oxidative stress • Reactive oxygen species generation • Depletion of the antioxidant, glutathione • Induces DNA damage at higher concentrations
Ãkerlund et al. [84]	Nickel (Ni) and nickel oxide (NiO)	Human bronchial epithelial cells (HBEC)	• Causes a release of inflammatory cytokines from exposed macrophages
Jiménez-Chávez et al. [85]	Titanium dioxide and Zinc oxide (TiO_2_ and ZnO)	Human alveolar epithelial cells (A549)	TiO_2_: • Shows a higher persistence in cell surface and uptake • Induces sustained inflammatory response (by means of TNF-Î ± , IL-10, and IL-6 release) • Induces reactive oxygen species generation ZnO: • Shows a modest response and low number in cell surface Both TiO_2_ and ZnO: • Concentration-dependent reduction in SP-A levels at 24 h of exposure to both TiO_2_ and ZnO • Cellular damage • Loss of lung function
Hussain & Garantziotis [86]	Cerium dioxide (CeO_2_)	Primary human monocytes	• Apoptosis (involving mitochondrial damage) • Causes a loss in membrane potential • Induces mitochondrial relocation of BAX • Induces modulation in autophagic events
Abudayyak et al. [87]	Bismuth Oxide—Bi (III) oxide (Bi_2_O_3_)	a) HepG2 human hepatocarcinoma cells (ATCC HB-8065) b) Caco-2 human colorectal adenocarcinoma cells (ATCC HTB-37) c) A549 human lung carcinoma cells (ATCC CCL-185)	• Induces apoptosis in HepG2 • Induces necrosis in A549 and Caco-2 cells • Causes significant changes in the levels of glutathione (GSH), malondialdehyde (MDA), and 8-hydroxydeoxyguanine (8-OHdG) in HepG2 and Caco-2 cells, except A549 cell
Fahmy et al. [88]	Copper/copper oxide (Cu/CuO)	Human diploid lung fibroblast normal cell lines (WI-38 cell) and human epithelial lung carcinoma cell lines (A549 cells)	• Suppresses proliferation and cell viability • Cause DNA damage • Induces generation of reactive oxygen species • Induces oxidative stress
Božinović et al. [89]	Molybdenum trioxide (MoO_3_)	Human keratinocyte (HaCaT) cell line	• Short exposure (up to 1 h) of keratinocytes to MoO_3_ has no significant impact on cell survival
Ahamed et al. [90]	Iron oxide (Fe_3_O_4_)	Skin epithelial A431 and lung epithelial A549 cell lines	• Induces dose-dependent cytotoxicity (indicated by reduction in cell viability lactate dehydrogenase leakage assays) • Induces dose-dependent oxidative stress • Induces reactive oxygen species • Induces lipid peroxidation • Causes DNA damage in high concentrations • Up-regulates the protein expression level of cleaved caspase-3
Pelclova et al. [91]	Titanium dioxide (TiO_2_)	Exhaled breath condensate (EBC) and urine	• Induces elevation of Leukotrienes (LT) levels • Induces inflammation and potential fibrotic changes in the lungs
Valdiglesias et al. [92]	Zinc oxide (ZnO)	Human neuroblastoma SHSY5Y cell line	• Apoptosis • Decreases cell viability • Induces cell cycle alterations • Induces micronuclei production • Induces H2AX phosphorylation and DNA damage
Verdon et al. [93]	Silver (Ag), Zinc oxide (ZnO), Copper oxide (CuO), Titanium dioxide (TiO_2_)	1) Human acute myeloid leukemia suspension cell line, HL-60 2) Primary neutrophils from human blood	• Ag and CuO nanoparticles stimulate neutrophil activation • TiO_2_ do not induce neutrophil response in either cell type • ZnO induces activation of HL-60 cells but does not activate primary cells
Siddiqui et al. [94]	Nickel oxide (NiO)	Cultured human airway epithelial (HEp-2) and human breast cancer (MCF-7) cells	• Apoptosis • Cytotoxicity • Reactive Oxygen species generation • Oxidative stress • generation and oxidative stress • Dietary antioxidant curcumin can effectively abrogate NiO NP-induced toxicity
Park et al. [95]	Cerium oxide (CeO_2_)	Human lung epithelial cells (BEAS-2B)	• Causes cell death • Reactive oxygen species production • Glutathione (GSH) decrease • Induces oxidative stress-related genes (e.g., heme oxygenase-1, catalase, glutathioneS-transferase, and thioredoxin reductase) • Apoptosis
Fakhar-e-Alam et al. [96]	Zinc oxide (ZnO)	Melanoma cells and human foreskin fibroblasts	• Induces reactive oxygen species production after UV-A-irradiation • Causes loss of mitochondrial membrane potential • Induces significant loss of cell viability
Hackenberg et al. [97]	zinc oxide (ZnO)	Human mucosa of the inferior nasal turbinate	• Repetitive exposure to low concentrations of ZnO-NPs results in persistent or ongoing DNA damage
Andujar et al. [98]	Welding-related NPs (essentially, Iron (Fe), Manganese (Mn), Chromium (Cr) oxide)	The lung of arc welders exposed to fume-issued NPs	• Induce the production of a pro-inflammatory secretome • All, but magnetite NPs, induce an increased migration of macrophages • NP-exposed macrophage secretome has no effect on human primary lung fibroblasts differentiation
Sharma et al. [99]	Zinc oxide (ZnO)	Human hepatocarcinoma cell line (HepG2)	• Decreases cell viability • Apoptosis • Induces DNA damage • Production of reactive oxygen species • Decreases mitochondria membrane potential • Activates JNK and p38 • Induces p53-Ser15 phosphorylation
Senapati et al. [100]	Zinc oxide (ZnO)	Human monocytic cell line, THP-1	• Induces oxidative and nitrosative stress • Causes an increase in inflammatory response (via activation of redox sensitive NF-kB and MAPK signalling pathways)
**Carbon-based Nanoparticles**			
Vlaanderen et al. [101]	Multi-walled carbon nanotubes (MWCNTs)	Breathing zone measurement of inhalable particulate matter, whole blood samples, and assessment of lung function of workers	• Significant upward trends for immune markers C–C motif ligand 20 (*p* = .005), basic fibroblast growth factor (*p* = .05), and soluble IL-1 receptor II (*p* = .0004) with increasing exposure to MWCNTs • Effect on lung health and immune system
Asghar et al. [102]	Carbon Nanotubes (CNT), Graphene Oxide (GO)	Human sperm	• Both SWCNT-COOH and reduced GO Causes no effect to sperm viability at lower concentrations • SWCNT-COOH generates significant reactive superoxide species at a higher concentration • Reduced graphene oxide does not initiate reactive species in human sperm
Periasamy et al. [103]	Carbon nanoparticles (CNPs)	Human mesenchymal stem cells (hMSCs)	• Reduces cell viability
Beard et al. [104]	Carbon nanotubes and nanofibers (CNT/F)	Sputum and blood	Inhalable rather than respirable CNT/F associated with: • Fibrosis • Inflammation • Oxidative stress • Cardiovascular biomarkers
Zhang et al. [105]	Single Wall Carbon nanotube (SWCNT)	Human ovarian cancer cell line OVCAR3	• Sensitises OVCAR3 cells to the chemotherapeutic compound paclitaxel (PTX) resulting in increased cell death • Apoptosis
de Gabory et al. [106]	Double-Walled Carbon Nanotubes (DWCNTs)	Human nasal epithelial cells (HNEpCs)	• Dose-dependent decrease in cell metabolic activity and cell growth • Stimulation of mucus production • Significant increase in Reactive Oxygen Species • Increased effect after 12-day exposure
Eldawud et al. [107]	Single Wall Carbon nanotubes (SWCNTs)	Immortalised human lung epithelial cell (BEAS-2B)	• Reducing cell viability • Changes cell structure, cycle and cell–cell interactions
Pacurari et al. [108]	Multi-walled carbon nanotubes (MWCNTs)	Human microvascular endothelial cells (HMVEC)	• Increase in endothelial monolayer permeability and migration in HMVEC • Induces endothelial cell permeability • Production of reactive oxygen species • Actin filament remodelling
Reamon-Buettner et al. [109]	Multi-walled carbon nanotubes (MWCNTs)	Human peritoneal mesothelial cells LP9	• Inhibition of cell division • Induces premature cellular senescence
Phuyal et al. [110]	Multi-walled carbon nanotubes (MWCNTs)	Human bronchial epithelial 3-KT (HBEC-3KT) cells	• Alters both the proteome and the lipidome profiles of the target epithelial cells in the lung
Witzmann & Monteiro-Riviere [111]	Multi-walled carbon nanotubes (MWCNTs)	Cryopreserved neonatal human epidermal keratinocytes	• Alters the protein expression in epithelial cells • Significant effect on the expression of cytoskeletal elements
Snyder et al. [112]	Multi-walled carbon nanotubes (MWCNTs)	Human bronchial epithelial primary cells	• Negatively impacts the ability of human airway epithelium to form a monolayer barrier • Altered cell morphology • Cytoskeletal disruption
Snyder et al. [113]	Multi-walled carbon nanotube (MWCNTs)	Human bronchial epithelial cells (BECs)	• Causes mitochondrial dysfunction that leads to mitophagy
Ghosh et al. [28]	Multi-walled carbon nanotubes (MWCNTs)	Allium cepa bulbs	• Cyto-genotoxicity • Induces significant DNA damage • Induces micronucleus formation • Chromosome aberration • Internucleosomal fragments formation, indicative of apoptotic cell death
Yu et al. [114]	Multi-walled carbon nanotubes (MWCNTs)	Immortalized human mesothelial cell line (Met-5A)	• Causes significant cytotoxic effects on Met-5A cells • Higher concentrations induce cellular membrane permeability and disturbance of mitochondrial metabolism • No significant toxic effect at low concentrations • Reactive oxygen species formation
Rizk et al. [115]	Multi-walled carbon nanotube (MWCNTs)	Normal human dermal fibroblast (NHDF) cells	• Induces induced massive loss of cell viability • DNA damage • Programmed cell death
Jos et al. [116]	Single wall carbon nanotubes (SWCNTs)	Human Caucasian colon adenocarcinoma (Caco-2) cell line	• Increase in Lactate dehydrogenase (LDH) leakage • Cytotoxicity • Protein content only modified at higher concentrations
Vankoningsloo et al. [117]	Multi-walled carbon nanotubes (MWCNTs)	1) Immortalised human keratinocytes (IHK) 2) SZ95 sebocytes 3) Reconstructed human epidermises (RHE)	• Induces cytotoxicity in human keratinocytes • No cytotoxic effects in SZ95 sebocytes or in stratified epidermises reconstructed in vitro
Herzog et al. [118]	Single-walled carbon nanotubes (SWCNTs), Carbon black (CB)	1) Human lung epithelial cells (A549) 2) Normal human primary bronchial epithelial cells (NHBE)	• Low oxidative stress • Cell responses are strongly dependent on the vehicle used for dispersion • The presence of dipalmitoyl phosphatidylcholine (DPPC) increased intracellular reactive oxygen species (ROS) formation
Müller et al. [119]	Single-walled carbon nanotubes (SWCNTs) and Titanium dioxide (TiO_2_)	Human epithelial lung cells (A549), human monocyte-derived macrophages (MDMs) and monocyte-derived dendritic cells (MDDCs)	• SWCNTs and TiO2 can penetrate into A549, MDMs, and MDDCs • Induces the production of reactive oxygen species
Baktur et al	Single-walled Carbon nanotubes (SWCNTs)	Human alveolar epithelial cells (A549)	• Enhances Interleukin-8 (IL-8) expression in the presence of serum • Induces changes in IL-8 expression
Basak et al. [120]	Multi-walled carbon nanotubes (MWCNTs) and TiO_2_ nanobelts (TiO_2_-NB)	Human colorectal adenocarcinoma cells	• Cytotoxicity
Patlolla et al. [121]	Multi-walled carbon nanotubes (MWCNTs)	Normal human dermal fibroblast cells (NHDF)	• Dose-dependent toxicity • Massive loss of cell viability through DNA damage • Cell death
Dahm et al. [122]	Carbon nanotubes and nanofibers (CNT/F)	Sputum samples Scanning electron microscopy (SEM)	• Industrial workers are exposed to the toxic effect of carbon nanotubes at the workplace
Fatkhutdinova et al. [123]	Multi-walled carbon nanotubes (MWCNTs)	Blood and sputum samples from workers	• Induction of pro-inflammatory cytokines (IL-6, TNF-α, and IL-1β) • Induction of KL-6 (a serological biomarker for interstitial lung disease) • Accumulation of inflammatory and fibrotic biomarkers in biofluids of workers manufacturing MWCNTs
Zhao et al. [124]	Multi-walled carbon nanotubes (MWCNTs) i.e., three commercially available MWCNTs, namely XFM4, XFM22, and XFM34 (diameters XFM4 < XFM22 < XFM34)	Human umbilical vein endothelial cells (HUVECs)	• XFM4 induced a significantly higher level of cytotoxicity than XFM22, and XFM34 • HUVECs internalized more XFM4 • XFM4 induces cytokine release, monocyte adhesion, and intracellular reactive oxygen species level • XFM4 exposure reduces the expression of autophagic genes autophagy-related 7 (ATG7), autophagy-related 12 (ATG12), and beclin 1 (BECN1) • Causes autophagy dysfunction and endoplasmic reticulum stress
Öner et al. [125]	Multi-walled carbon nanotubes (MWCNTs) and Single-walled carbon nanotubes (SWCNTs)	Human bronchial epithelial cells (16HBE)	• MWCNTs induce a single hypomethylation at a CpG site and gene promoter region • No change in DNA methylation after the recovery period for MWCNTs • SWCNTs or amosite induce hypermethylation at CpG sites after sub-chronic exposure
Luanpitpong et al. [126]	Carbon nanotubes (CNT)	Non-tumorigenic human lung epithelial cells	• Induces Cancer Stem-like cells (CSC) in lung epithelial cells • Induces specific stem cell surface markers CD24 low and CD133 high that are associated with SWCNT-induced CSC formation and tumorigenesis
Shvedova et al. [127]	Carbon nanotubes (CNT)—Multi-walled carbon nanotubes (MWCNTs)	Air Sample Peripheral whole blood	• Causes significant changes in the non-coding RNAs (ncRNA) and coding messenger RNAs (mRNA) expression profiles • Cell cycle regulation/progression/control • Apoptosis and proliferation • Potential to trigger pulmonary and cardiovascular effects • Potential to induce carcinogenic outcomes in humans
Domenech et al. [128]	Carbon-based nanoparticles: Graphene oxide (GO), Graphene nanoplatelets (GNPs)	Human colorectal adenocarcinomas (Caco-2, ATCC HTB-37)	• No oxidative damage induction was detected, either by the DCFH-DA assay or the FPG enzyme in the comet assay • Both GO and GNPs induce DNA breaks • Induces weak anti-inflammatory response
Wang et al. [129]	Single-walled carbon nanotubes (SWCNT) and multi-walled carbon nanotubes (MWCNTs)	Primary human Small Airway epithelial Cells (SAECs)	• Increases cell proliferation • Induces anchorage-independent growth • Causes cell invasion and angiogenesis
Mukherjee et al. [130]	Graphene oxide (GO)	human bronchial epithelium (BEAS-2B) cells	• Causes mitochondrial dysfunction after a 48-h exposure • Causes engagement of apoptosis pathways after longer exposure periods (i.e., 28 days) • Causes down regulation of genes belonging to the inhibitor of apoptosis protein (IAP) family
Pérez et al. [131]	Reduced graphene oxide (rGO)	Human airway epithelial (BEAS-2B) cells	• Medium-term rGO exposure does not have significant effects on the DNA methylation patterns of human lung epithelial cells
Xu et al. [132]	Single-walled carbon nanotubes (SWCNT)	Pulmonary surfactant monolayer (PSM)	• Inhalation toxicity of SWCNTs is largely affected by their lengths • Short SWCNTs increases inflammatory response • Longer SWCNTs causes severe lipid depletion and PSM-rigidifying effect
Gasser et al. [133]	Multi-walled carbon nanotubes (MWCNTs)	Human monocyte derived macrophages (MDM) monocultures A sophisticated in vitro model of the human epithelial airway barrier	• Increases reactive oxygen species levels • Decreases intracellular glutathione depletion in MDM • Decreases the release of Tumour necrosis factor alpha (TNF-Î ±) • Induces apoptosis • Increases the release of the release of Interleukin-8 (IL-8)
Di Cristo et al. [134]	Graphene Oxide (GO)	EpiAirway™ tissues (AIR-100, PE6-5), a 3D mucociliary tissue model of the primary human bronchial epithelium	• Elicits proinflammatory response after 2 weeks exposure • Causes moderate barrier impairment • Induces autophagosome accumulation (resulting from blockade of autophagy flux) • Prolonged exposures increase the risk of pulmonary infections and/or lung diseases
Chortarea et al. [135]	Carbon nanotubes (CNTs)	Human (alveolar) epithelial A549 cell line with human monocyte-derived dendritic cells (MDDCs) and macrophages (MDMs)	• Repeated exposures to lung cell cultures at the Air–Liquid Interface, elicit a limited biological impact over a three-day period
Lee et al. [26]	Graphene oxide (GO)	Workplace air samples	• Minimum release of graphene or other particles during manufacturing based on real-time aerosol monitoring • Negligible exposure to graphene based on personal and area sampling for the Total Suspended Particles (TSP) and elemental carbon (EC)
**Both Inorganic-based and Carbon-based Nanoparticles**			
Phuyal et al. [21]	Titanium dioxide (TiO_2_) and multi-walled carbon nanotubes (MWCNTs)	Human bronchial epithelial (HBEC-3KT) cell line	• Low cytotoxicity in short-term tests • Cell proliferation affected in long-term exposure
Shalini et al. [22]	Zinc oxide (ZnO) and nanorods	Human peripheral blood lymphocytes (HPBL)	• Genotoxicity in smaller ZnO NPs • Cytotoxic effect in larger microparticles and microrods • Higher level of oxidative potential and reactive oxygen species generation capacity in ZnO NPs and nanorods
Simon-Deckers et al. [23]	Aluminium oxide (Al_2_O_3_), Titanium dioxide – (TiO_2_), Multi-walled carbon nanotubes (MWCNTs)	A549 human type II lung epithelium cell line	• Carbon nanotubes are more toxic than metal oxide NPs • Both nanotubes and NPs rapidly enter into cells, and distribute in the cytoplasm and intracellular vesicles
**Inorganic-based and Polymer Nanoparticles**			
Setyawati et al. [24]	Titanium dioxide (TiO_2_), Terbium-doped gadolinium oxide (Tb-Gd_2_O_3_), and Poly (lactic-co-glycolic acid) (PLGA)	Human neonatal foreskin fibroblast cell line (BJ)	TiO_2_ and Tb-Gd_2_O_3_: • Dose-dependent cytotoxicity • Promotes genotoxicity via DNA damage PLGA nanoparticles: • Did not induce significant cytotoxic or genotoxic effects on BJ
**Polymer Nanoparticles**			
Nishu et al. [25]., 2020	Poly lactic-co-glycolic acid (PLGA)	Nitrosomonas europaea KCTC 12270 bacterium Nitrospira moscoviensis bacterium	• Reduce nitrification in both cultures of nitrifying strains and in microbial communities in soil samples

## Discussion

The objective of this scoping review was to ascertain the distribution of the current literature on the human and environmental impacts of nanoparticles. Specifically, in this review, we synthesised evidence regarding the exposure pathways and types of nanoparticles that have been researched and the ones that have not, as well as the biomarkers that have been used in assessing human and environmental impact of exposure to nanoparticles.

### Characteristics of included studies

While the majority of studies originated from Europe and Central Asia, the United States of America (USA) alone recorded the highest number of publications. This finding is not surprising, as the USA has continuously fostered the development of nanotechnology through significant investments in research and development in this area. In 2016, the USA was projected to account for almost one-third of total global nanotechnology research funding [136]. Moreover, the USA and the European Union have over the years taken a committed approach towards enhancing the health and safety of nanoparticles [137]. As part of this commitment, annual meetings are held, where researchers discuss topics relating to nano-safety, as well as funding priorities and research needs.

While there have been some investments in nanotechnology research in African countries (including Egypt and South Africa), a recent publication by the United Nations Economic Commission for Africa (UNECA) indicates that the African continent, relative to other continents, is lagging behind with regards to nanotechnology research [138]. This assertion is consistent with the findings of this review, which found only one study originating from North Africa (Egypt), with no study conducted in Sub-Saharan Africa.

Over the past two decades, there have been increasing public awareness of nanotechnology and a growing concern about its commercial applications [139]. This has led to rapidly increasing scientific publications in this field, especially from early 2000s [140]. It is, therefore, not surprising that the studies included in this scoping review were published from the year 2006. Indeed, a literature search of nanotechnology publications by Huang et al. [140] revealed over 50,000 publications for the year 2006.

Although the included studies investigated a wide range of nanoparticles, most of them focused on inorganic-based nanoparticles (e.g., zinc oxide, titanium dioxide, copper oxide, and silica), followed by carbon-based nanoparticles (e.g., carbon-nanotubes, fullerenes, and graphene) (Table 3). This finding is consistent with previous reviews that have reported extensive investigation into the impact of inorganic-based and/or carbon-based nanoparticles [141, 142]. These nanoparticles may have gained attention due to their extensive production and usage. In addition to their use for cancer treatment, inorganic and carbon-based nanoparticles provide significant benefits in photothermal therapy, diagnosis, tissue engineering, imaging contrast agents, and sensing applications [143]. This is due to their unique physical and chemical properties (such as electrical, thermal, structural, mechanical, and optical diversity), which make them stronger, flexible, and more electrically conductible towards several biological entities [141, 144]. The advantages of, for example inorganic-based nanoparticles, including their high reactivity, small size and good capacity have been found to induce adverse harmful effects in both humans and the environment.

In this review, a number of approaches were used by included studies to assess the toxicity of nanoparticles. However, the majority of the studies applied the in vitro method, perhaps because in vitro studies are time saving and cost-effective. Nonetheless, the in vitro approach has been criticised by researchers (e.g., Bahadar et al. [145]) for producing varying results in different laboratories.

The included studies used differing methods in assessing cytotoxicity and genotoxicity: cell membrane integrity was assessed with Lactate dehydrogenase (LDH) assays [44, 57, 116]; cell viability was assessed using tetrazolium reduction assays [82, 83, 90, 116]; apoptosis was assessed using immunohistochemistry biomarkers [60, 65, 86]; electron microscopy was used to assess intracellular localisation of nanoparticles [34, 106]; and cell inflammation was estimated using chemokines biomarkers (i.e., IL-8, TNF- α, and IL-6) [146]. Compounds such as MTT, XTT, MTS, and WST-1 are used to detect viable cells [147]. However, in the current review, most of the studies employed MTT tetrazolium assays for investigating cell toxicity [47, 49, 50, 58, 116]. Similar findings have been reported by Bahadar et al. [145] who conducted a review on the toxicity of nanoparticles.

### The human impact of nanoparticles

Most of the studies in this review focused on assessing the characteristics of nanoparticles, as well as the impact of nanoparticles on, particularly, human health. In recent years, there have been promising results from the application of nanoparticles to human health, especially in cancer treatment. This is due to the potential of nanoparticles to provide innovative solutions to curb the limitations of traditional treatment methods, including radiotherapy and chemotherapy [148]. Relative to conventional cancer treatment methods, nanoparticle-based drug delivery systems have been shown to have significant advantages in a) drug resistance, b) correctly targeting tumour cells, c) having good pharmacokinetics, and d) reduction of treatment side effects [149]. Notwithstanding these benefits, however, nanoparticles have potential harmful effects, and there are controversies about their safe use in humans [139]. This has undoubtedly led to the rapidly growing number of studies investigating the human health impact of nanoparticles, as was revealed in this review.

The majority of the studies (n = 90) in this review used immortalised cell lines as the biomarker for assessing human health impact of nanoparticles, and only 22 studies used primary cells as biomarkers. Immortalised cell lines have mostly been used for nano-safety studies because, relative to primary cells, they are generally less expensive, readily accessible, and easier to cultivate [150]. However, the type of cell that is used as biomarker for nano-safety studies is of great importance since this may have an impact on the general outcome of studies [151]. Cancer cell lines, for example, have a disturbed anti-apoptotic balance, and have undergone transformation in metabolism, which impacts their ability to sustain their high rate of proliferation [152]. As such, using these cells may have an impact on study findings. Nonetheless, the use of primary cells in nano-safety studies, are not without limitations. Primary cells have limited lifespan in vitro and can suffer from clonal changes.

In using immortalised cell lines, several studies [153, 154]) have reported variations in findings regarding nanoparticle-induced effects in cell lines obtained from different species or tissues. For example, Zhang et al. [153] and Mukherjee et al. [154] investigated the effect of exposure to silver nanoparticle on mammalian cells. Zhang et al. [153] used epithelial cells and microphages, and Mukherjee et al. [154] used the human dermal and cervical cell lines as biomarkers. Mukherjee et al. [154] reported nanoparticle-induced cytotoxicity such as elevated levels of oxidative stress, cell membrane damage, and glutathione depletion, whereas Zhang et al. [153] reported effects including changes in antioxidant defence and metallothionein. Moreover, while Ekstrand-Hammarstrom et al. [155] and Kermanizadeh et al. [156] have compared the effect of nanoparticles on immortalised cell lines versus primary cells of the same species and tissues, available data regarding the relative effectiveness of these two types of cells are unclear. Therefore, it is difficult to make explicit conclusions as to which of these two types of cells can be used as a reliable biomarker for nano-safety studies.

This review has revealed that humans are exposed to nanoparticles through inhalation, ingestion, or dermal route. After their exposure, nanoparticles induce toxic effects such as production of oxidative stress at the exposure site, inflammation, DNA damage, and cell death [87, 88]. For instance, exposure of human neuroblastoma (Sh-sy5y) cells to inorganic nanoparticles, such as titanium dioxide, silica dioxide, and silver are associated with induction of neurotoxicity, membrane damage, reaction oxygen specie formation, decrease in cell viability, and autophagy dysfunction [40]. Similarly, exposure to carbon-based nanoparticles such as single and multi-walled carbon nanotubes reduce cell viability, as well as induce changes in cell structure, cell cycle, and cell-to-cell interactions in human lung epithelial cells (BEAS-2B) [107].

### The environmental impact of nanoparticles

The findings of this scoping review indicate a gap in the literature regarding environmental impact of nanoparticles. Out of the 117 included studies, only 5 had assessed the environmental impact of exposure to nanoparticles. This significant gap in the scientific literature has been highlighted by authors such as Bundschuh et al. [157]. The growing production and usage of nanoparticles has undoubtedly led to a diversification of emission sources into both the aquatic and soil environment. Nanoparticles enter the environment mainly through three emission scenarios: a) released during production of nano-enabled products and raw materials, b) during application, and c) following disposal of products containing nanoparticles [158]. These emissions occur either indirectly through systems such as landfills or wastewater treatment plants, or directly to the environment. Nonetheless, nanoparticles are mostly released during the application phase and following disposal [159]. Indeed, during production, only about 2% of the production volume is emitted [160]. The studies in this review used biomarkers such as soil samples and soybean seeds, zebrafish larvae, fish, and Daphnia magna neonates. This finding is in line with a previous review by Bundschuh et al. [157], which explored the effects of nanoparticles on the environment.

### Limitations of the review

In this review, every effort was made to reduce bias. The search strategy was developed by experts of the review team with many years of experience in conducting systematic/scoping reviews. A comprehensive search of multiple relevant databases and other resources was conducted by one review author (EAK) and a rerun of the searches was done after 4 weeks of the initial search. Two authors (EAK and RF or PB and SH) independently screened the search results, and disagreements between reviewers were resolved by FVZ or TP.

The main limitation of this review is that the searches were limited to studies published in the English language. This may have led to the exclusion of potentially relevant papers published in other languages. Also, searches were restricted to studies published from the year 2000, which may have led to the omission of potentially relevant papers.

## Conclusions

This review has provided an extensive synthesis of the current literature on the effects of nanoparticles on human health and the environment. The review has shown that while nanoparticles are beneficial in a range of applications, they pose significant threats to humans and the environment. Through the use of several biological models and biomarkers (e.g., human bronchial epithelial cells (Beas-2), soil samples, and soybean seeds), the included studies revealed the toxic effects of nanoparticles, with the most investigated nanoparticles being Zinc Oxide, MWCNTs, Titanium Dioxide, Cerium Oxide, SWCNTs, Ferric Oxide, and Silicon Dioxide. The main health impacts of nanoparticles identified in this review are decreased cell viability, cell death, reactive oxygen species generation, production of oxidative stress (dose-dependent), DNA damage, apoptosis, and induction of inflammatory responses.

This review has revealed a significant gap in the scientific literature regarding environmental impact of nanoparticles of all types. Future studies should be directed at investigating the impact of the various types of nanoparticles on the aquatic, terrestrial, and soil environment. The findings from this review have also shown limited data regarding the relative effectiveness of immortalised cell lines and primary cells as biomarkers in nano-safety studies. Future research should focus on evaluating the effectiveness of these two types of cells, in order to determine the cell that can be used as a reliable biomarker for nano-safety studies. There is also the need for future studies in this area to focus on exploring the toxic effects of Platinum, Gold, Magnesium Oxide, Molybdenum Trioxide, Tungsten trioxide, and Carbon Black nanoparticles, as findings from this review has shown that these nanoparticles are least researched. The findings of this review will be useful to policy makers and stakeholders in assessing the potential effects of nanoparticles.

## Supplementary Information

**Additional file 1.** MEDLINE Search History.

## Data Availability

All data generated or analysed during this study are included in this published article and in the presented supplementary material file.
