# Supplementary material for: Human and environmental impacts of nanoparticles: a scoping review of the current literature
Source: BMC Public Health. 2023 Jun 3;23:1059. doi: 10.1186/s12889-023-15958-4 (PMC10239112; doi:10.1186/s12889-023-15958-4)
Supplement: Supplementary file 1 — Additional file 1. MEDLINE Search History. [file 12889_2023_15958_MOESM1_ESM.docx]

**Title: Human and environmental impacts of nanoparticles: a scoping review of the current literature**

**Author Details:**

Dr Elizabeth Adjoa Kumah^1^

Raoul Djou Fopa^2^

Saeed Harati^2^

Dr Paul Boadu^3^

Dr Fatemeh Vida Zohoori, Professor^4^

Dr Tannaz Pak^2^

^1^Depeartment of International Public Health, Liverpool School of Tropical Medicine, Liverpool, UK

^2^School of Computing, Engineering & Digital Technologies, Teesside University, Middlesbrough, UK

^3^ Department of Health Services Research and Policy, London School of Hygiene and Tropical Medicine, London, UK

^4^School of Health and Life Sciences, Teesside University, Middlesbrough, UK

**MEDLINE Search History**

| **#** | **Query** | **Limiters/Expanders** | **Last Run** | **Results/Number of Hits** |
| --- | --- | --- | --- | --- |
| S12 | S4 AND S8 | Limiters - Published Date: 20000101-20211231; Peer Reviewed Expanders - Apply equivalent subjects Search modes - Boolean/Phrase | Interface - EBSCOhost Research Databases Search Screen - Advanced Search Database - CINAHL Plus with Full Text | 1,381 |
| S11 | S4 AND S8 | Limiters - Published Date: 20000101-20211231 Expanders - Apply equivalent subjects Search modes - Boolean/Phrase | Interface - EBSCOhost Research Databases Search Screen - Advanced Search Database - CINAHL Plus with Full Text | 1,450 |
| S10 | S4 AND S8 | Expanders - Apply equivalent subjects Search modes - Boolean/Phrase | Interface - EBSCOhost Research Databases Search Screen - Advanced Search Database - CINAHL Plus with Full Text | 1,474 |
| S9 | S4 AND S8 | Expanders - Apply equivalent subjects Search modes - Boolean/Phrase | Interface - EBSCOhost Research Databases Search Screen - Advanced Search Database - CINAHL Plus with Full Text | 1,474 |
| S8 | S5 OR S6 OR S7 | Expanders - Apply equivalent subjects Search modes - Boolean/Phrase | Interface - EBSCOhost Research Databases Search Screen - Advanced Search Database - CINAHL Plus with Full Text | 2,274,604 |
| S7 | nanotoxic* | Expanders - Apply equivalent subjects Search modes - Boolean/Phrase | Interface - EBSCOhost Research Databases Search Screen - Advanced Search Database - CINAHL Plus with Full Text | 46 |
| S6 | health* | Expanders - Apply equivalent subjects Search modes - Boolean/Phrase | Interface - EBSCOhost Research Databases Search Screen - Advanced Search Database - CINAHL Plus with Full Text | 2,210,239 |
| S5 | toxic* | Expanders - Apply equivalent subjects Search modes - Boolean/Phrase | Interface - EBSCOhost Research Databases Search Screen - Advanced Search Database - CINAHL Plus with Full Text | 77,584 |
| S4 | S1 OR S2 OR S3 | Expanders - Apply equivalent subjects Search modes - Boolean/Phrase | Interface - EBSCOhost Research Databases Search Screen - Advanced Search Database - CINAHL Plus with Full Text | 5,848 |
| S3 | nanostructure* | Expanders - Apply equivalent subjects Search modes - Boolean/Phrase | Interface - EBSCOhost Research Databases Search Screen - Advanced Search Database - CINAHL Plus with Full Text | 764 |
| S2 | nanoparticle* | Expanders - Apply equivalent subjects Search modes - Boolean/Phrase | Interface - EBSCOhost Research Databases Search Screen - Advanced Search Database - CINAHL Plus with Full Text | 5,061 |
| S1 | nanomaterial* | Expanders - Apply equivalent subjects Search modes - Boolean/Phrase | Interface - EBSCOhost Research Databases Search Screen - Advanced Search Database - CINAHL Plus with Full Text | 820 |
